# Global Forecasting Confirmed and Fatal Cases of COVID-19 Outbreak Using Autoregressive Integrated Moving Average Model

**DOI:** 10.3389/fpubh.2020.580327

**Published:** 2020-10-29

**Authors:** Debabrata Dansana, Raghvendra Kumar, Janmejoy Das Adhikari, Mans Mohapatra, Rohit Sharma, Ishaani Priyadarshini, Dac-Nhuong Le

**Affiliations:** ^1^Department of Computer Science and Engineering, GIET University, Gunupur, India; ^2^Department of Electronics & Communication Engineering, SRM Institute of Science and Technology, Ghaziabad, India; ^3^Department of Electrical and Computer Engineering, University of Delaware, Newark, DE, United States; ^4^Institute of Research and Development, Duy Tan University, Da Nang, Vietnam; ^5^Faculty of Information Technology, Duy Tan University, Da Nang, Vietnam

**Keywords:** COVID-19, ARIMA, forecasting, global pandemic, time series analysis

## Abstract

The world health organization (WHO) formally proclaimed the novel coronavirus, called COVID-19, a worldwide pandemic on March 11 2020. In December 2019, COVID-19 was first identified in Wuhan city, China, and now coronavirus has spread across various nations infecting more than 198 countries. As the cities around China started getting contaminated, the number of cases increased exponentially. As of March 18 2020, the number of confirmed cases worldwide was more than 250,000, and Asia alone had more than 81,000 cases. The proposed model uses time series analysis to forecast the outbreak of COVID-19 around the world in the upcoming days by using an autoregressive integrated moving average (ARIMA). We analyze data from February 1 2020 to April 1 2020. The result shows that 120,000 confirmed fatal cases are forecasted using ARIMA by April 1 2020. Moreover, we have also evaluated the total confirmed cases, the total fatal cases, autocorrelation function, and white noise time-series for both confirmed cases and fatalities in the COVID-19 outbreak.

## Introduction

The first case of the virus came to light in Wuhan city of China in November 2019. The population of Wuhan city is nearly 11 million, and it connects to many major cities in China. The number of cases changed to dozens and then hundreds by the end of December. Medical experts first suspected that it was viral pneumonia, which could not be cured with conventional medicines. Ever since the virus first started to infect people, it has continued to spread and affect thousands of people (1). Further, every patient infected with this virus was infecting two or three people ahead of them. Until December 30 2019, no information was released from China regarding the deadly virus. Finally, in December 2020, officials from the public health department of China informed the World Health Organization about the medical issue that affected people in Hubei Province, China. The infection was described as a pneumonia-like ailment in humans and caused by a coronavirus, an extreme group of pathogens. Coronaviruses are known to spread among people, mice, winged creatures, bats, domesticated animals, and other wild creatures (2–4). In December 2019, the WHO was alerted by China to certain occurrences of a respiratory infection associated with specific people who had visited the seafood market in Wuhan city (5). Wuhan experienced the spread of a coronavirus, called Coronavirus Disease-19 (COVID-19). In (6), the author presumed that COVID-19 likely started in bats, since it is progressively like two bat-determined coronavirus strains. However, the origin of the COVID-19 has not yet been confirmed at this point, and it requires more investigation. In 2003 and 2012, the Middle East Respiratory Syndrome (MERS) coronavirus and Severe Acute Respiratory Syndrome (SARS) coronavirus were found to be zoonotic such that they may be transmitted among animals and humans (7). COVID-19 is the third profoundly pathogenic human coronavirus that has been identified over the most recent two decades. The individual-to-individual transmission has been depicted both in emergency clinics and family settings (8). Therefore, it is necessary to forestall any further spread in the general society and in human services settings. COVID-19 transmission through tainted dry surfaces makes it even easier to transmit. Hence self-immunization of the mucous layers of the nose, eyes, or mouth has been proposed (9–11). Biocidal products like hydrogen peroxide, alcohols, sodium hypochlorite, and benzalkonium chloride are being utilized worldwide for sanitization purposes, especially in social settings (12).

As of March 25 2020, 18,295 individuals had died from COVID-19 infection, while 107,089 patients recovered. As per the WHO, there were more than 411,242 confirmed cases worldwide, with the majority of revealed cases in Wuhan city. This led to Wuhan placing a citywide lockdown on January 23 2020, in which no individuals were permitted to enter or leave. The officials temporarily suspended all accessible transportation, including trains, metro, air terminals, and public vehicles to avoid the spread of COVID-19. Also, a few urban areas in Hubei territory were put under lockdown. One of the challenges posed by COVID-19 is its quarantine period, which is as long as 2–14 days (13), and during this period, it can spread to others. Besides, in (14), it is mentioned that the alone time may range from 0 to 24 days depending upon the situation of the patient. The spread of such sickness is unbelievably dangerous. It requires continuously extraordinary blueprints and plans, which have been executed in different Chinese urban districts, particularly in the Hubei area. Hence it is indispensable to explore the number of confirmed cases at this time to start the vital assertion plans. The main contribution of this research work is the use of an ARIMA model (15), which is capable of forecasting the global pandemic COVID-19 using the dataset, as shown in Table 1. The main contributions are per the following:

- We used a proficient forecasting model to find the confirmed cases of COVID-19 dependent on recently confirmed cases.
- An ARIMA model was used to forecast the exact confirmed fatalities of the coronavirus outbreak from February 1 2020 to April 1 2020.
- We evaluated total confirmed cases, total fatalities, confirmed cases concerning fatal cases, Q-Q plot of confirmed and fatal cases, white noise confirmed cases vs. fatal cases, and lastly the autocorrelation function between confirmed and fatalities cases.

The rest of this research has been organized as follows: section Literature Survey provides a survey of the previous work. Dataset description and ARIMA model are discussed in section Material and Methods. The results and their analysis are illustrated in section Result and Discussion. Finally, we conclude the paper in section Conclusion.

**Table 1:** Dataset used in this study.

**Province state**	**Country region**	**Date (M/D/Y)**	**Confirmed cases**	**Fatalities**
Hubei	China	1/22/2020	444	17
Hubei	China	1/23/2020	444	17
Hubei	China	1/24/2020	549	24
Hubei	China	1/25/2020	761	40
Hubei	China	1/26/2020	1,058	52
Hubei	China	1/27/2020	1,423	76
Hubei	China	1/28/2020	3,554	125
Hubei	China	1/29/2020	3,554	125
Hubei	China	1/30/2020	4,903	162
Hubei	China	1/31/2020	5,806	204
Hubei	China	2/1/2020	7,153	249
Hubei	China	2/2/2020	11,177	350
Hubei	China	2/3/2020	13,522	414
Hubei	China	2/4/2020	16,678	479
Hubei	China	2/5/2020	19,665	549

## Literature Survey

Existing work has been conducted in the past to evaluate estimation problems, like an adaptive neuro-fuzzy inference system (ANFIS) (16), which is applied extensively in the time course of action desire and envisioning issues, and it indicated that there was incredible execution in the present application. It offers adaptability for handling non-linearity in time series data, by combining an artificial neural network (ANN) and a fuzzy approach. ARIMA models applied to historical hemorrhagic fever with renal syndrome (HFRS) occurrence information are a significant device for HFRS observation in China. Chinese HFRS information from 1975 to 2008 was taken into account for fitting the ARIMA model. Akaike information criterion (AIC) and the Ljung-Box test have been relied on for assessing the developed models. Along these lines, the fitted ARIMA model was applied to get the suited HFRS frequency from 1978 to 2008 and appeared differently concerning the corresponding observed values (17). This paper highlights the significance of embracing dynamic modeling approaches, proposes difficulties for performing model determination across long time periods, and relates comprehensively to the predictability of complex adaptive systems. (18) introduced an ensemble model for sequential forecasting using a frequent computational bootstrap approach to evaluate the Ebola outbreak and generated short-term forecasts of the epidemic outbreak by combining two models, the generalized-growth model (GGM) and the generalized-logistic model (GLM) (19). The seasonal autoregressive-integrated moving average (SARIMA) model is used to forecast monthly cases of hand, foot, and mouth disease (HFMD) in China (20). A short-term forecast of incidence in China has been done by applying ARIMA and exponential smoothing (ETS) that analyzed data from the Chinese Center for Disease Control and Prevention between 2005 and 2006 (21).

Ture and Kurt (22) proposed a comparative study among different types of time series methods to forecast Hepatitis A virus (HAV) infection. The methods considered were the ANN algorithm, radial basis function (RBF), time-delay neural networks (TDNN), and the ARIMA model, where the ANN algorithm was found to be more accurate than the others. In paper (15), the authors proposed to apply a susceptible–infectious–recovered–susceptible (SIRS) mathematical model estimating model dependence on gathering alteration Kalman channels for occasional flare-ups of flu. They assessed the proposed model utilizing the flu season information of New York City for a long period (2003–2008). Massad et al. (23), proposed a numerical model to break down and gauge the disease of the SARS epidemic to survey the viability of these techniques. Here the author worked to determine 13 years of time series data. In another work, Shaman et al. (24) formulated three scenarios based on a hypothesis about under-reporting of EVD cases and the EVD case fatality ratio using a standard life table technique to calculate the life expectancy of Ebola virus disease (EVD) patients in a couple of African countries.

On the basis of the existing studies, in this paper study, the ARIMA model was used for time series analysis to either get comprehensive information or to anticipate future qualities. This model is applicable *in situ*ations where information may be non-fixed. Non-fixed practices can be patterns, a cycle, random walks, or mixes of the three. Non-fixed information focuses are unusual and can not be displayed or estimated. An investigation utilizing non-fixed time arrangement information focuses may not be fitting as it might show the connection between two factors where one does not exist. To get predictable, reliable outcomes, the non-fixed information should be changed into fixed information. The non-fixed procedure and the fixed procedure around a consistent long haul has a steady difference autonomous of time.

## Materials and Methods

### Dataset

The dataset considered for the study has been collected from relevant sources (https://www.kaggle.com/c/covid19-global-forecasting-week-2/data). It contains the day to day confirmed cases from all over the world between January 22 2020 and March 31 2020. An overview of the dataset has been shown in Table 1. The dataset consists of a total of 22,032 columns and 7 rows. The COVID-19 dataset also includes 5 attributes, i.e., id, prov_state, country_region, confirmed case, and fatal case. The data is in the form of time-series data points. Time-series is a sequence of information that describes the time period of each value. Generally, time-series data used for analysis and forecasting the future is based on historical data. Time-series data determines the stability of a situation over time and efficiency portfolios. Time-series datasets are time-dependent because values for every period are affected by outside factors and the values of the past period. During the dataset loading operation, we considered the date as our index column. Therefore, the date column is no longer a feature for us. This is because time-series data perform tasks related to the date. That is why it is the most used parameter in our methodology (Table 1).

### Autoregressive Integrated Moving Average (ARIMA)

ARIMA is a famous and adaptable class of forecasting models that uses recorded data to make estimations. This model is an essential forecasting technique that can serve as a starting point for progressively complex models (15). It works effectively when the information displays a steady or predictable example after some time with a base measure of anomalies. The ARIMA approach endeavors to portray developments in a stationary time series as an element of what is designated as “autoregressive and moving normal” parameters. These are alluded to as autoregressive parameters and moving average (MA) parameters. We accept time is a discrete variable, *Z*_*t*_ shows the observation at time *t* and t demonstrates the zero-mean random noise term at time t. The MA(*n*) (moving average) model uses this procedure:

(1) $$\begin{array}{r} Zt=\overset{n}{\underset{i=1}{\sum }}\gamma_{\text{i}}{€}_{\text{t}-1}+{€}_{\text{t}} \end{array}$$

where **γ**_***i***_ denotes coefficient, similar to MA(*n*) models, autoregression model, denoted by AR(*m*),

(2) $$\begin{array}{r} Zt=\overset{m}{\underset{i=1}{\sum }}\delta_{\text{i}}Z_{\text{t}-1}+{€}_{\text{t}} \end{array}$$

*Zt* is a noisy linear combination of the previously taken *m* observations. An increasingly advanced model is the ARIMA (*m, n*), mix of AR(*m*), and MA(*n*) with a reduced structure and gives an adaptable demonstrating system. This model expects that Zt is created through the formula:

(3) $$\begin{array}{r} Z_{\text{t}}=\overset{n}{\underset{i=1}{\sum }}\gamma_{\text{i}}{€}_{\text{t}-1}+\overset{m}{\underset{i=1}{\sum }}\delta_{\text{i}}Z_{\text{t}-1}+{€}_{\text{t},} \end{array}$$

where *t* is the zero-mean noise term. On the off chance that we are adding imperative to the AR(*m*) part, it ensures a stationary process. A fixed and invertible ARIMA (*m, n*) model may be depicted either as an infinite AR model (AR(∞)) or an infinite MA model (MA(∞)). For the ARIMA model, one can compute the first-order differences of Zt by ∇Zt= Z_t_-Z_t−1_ and second-order differences of Zt by ∇2Zt= ∇Zt–∇Zt-1 such that the sequence of ∇dZt satisfies an ARIMA (*m, n*). We state that the sequence of Zt satisfies the ARIMA (*m, d, n*).

(4) $$\begin{array}{r} \nabla^{d}Z_{t}=\overset{n}{\underset{i=1}{\sum }}\gamma_{\text{i}}{€}_{\text{t}-1}+\overset{m}{\underset{i=1}{\sum }}\delta i\nabla^{d}Zt_{-1}+{€}_{\text{t},} \end{array}$$

which are specified by three order parameters terms *m, d, n* with specific weights vector δ∈R *m* and γ∈R*n*. Forecasting with ARIMA *(m, d, n)* is an inversion of the differential equation. Assuming the time-series sequence *Zt* fulfills ARIMA *(m, d, n)*, one can predict the *d-*th order differential of observation at time *t* + *1* as ∇^*d*^$Z_{\text{t}+1}^{\sim }$ and then predict the observation at time *t* + 1 as $Z_{t}^{\sim }$:

(5) $$\begin{array}{r} Z_{t}^{\sim }=\nabla^{d}Z_{t}^{\sim }+\overset{d-1}{\underset{i=0}{\sum }}.\nabla^{i}Z_{t-1} \end{array}$$

## Result and Discussion

**Figure 1:**
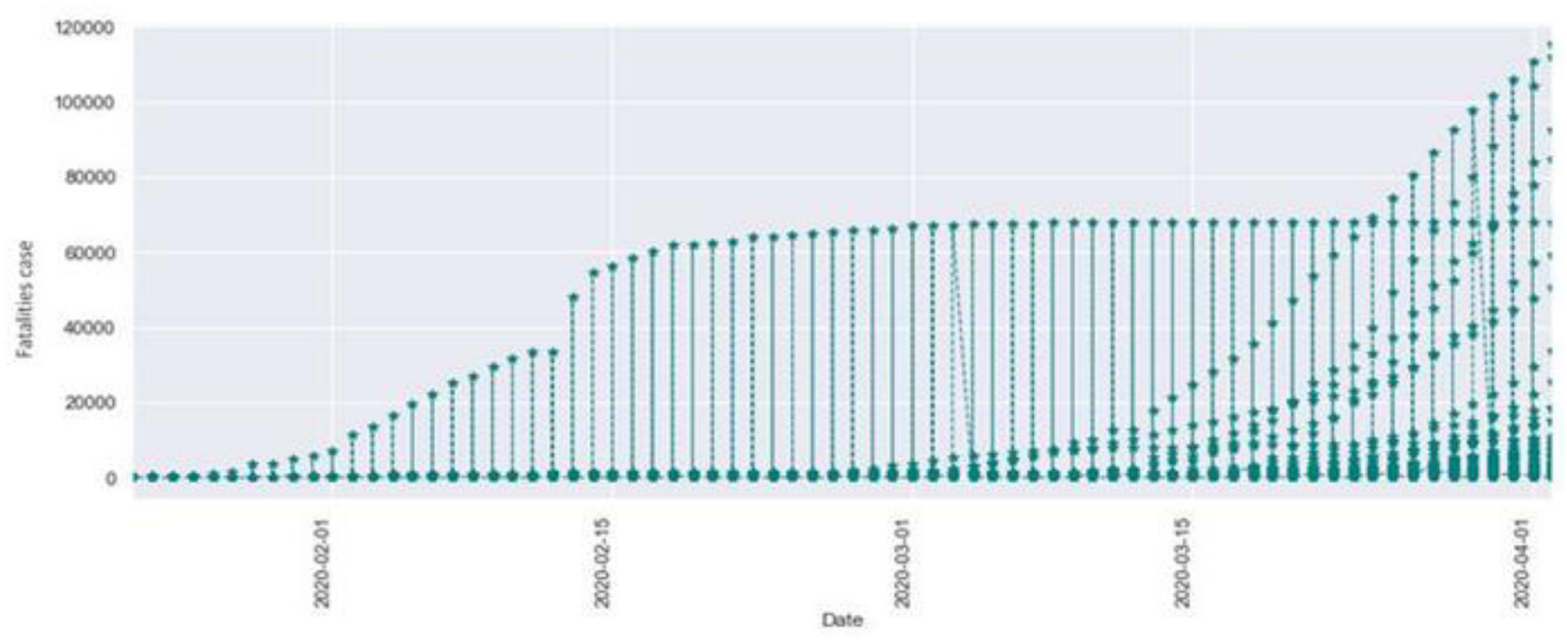
Total confirmed cases of COVID-19.

For this study, data were analyzed using a python library called matplotlib. It is a popular package for plotting 2D data. This library has been used to derive the line charts of the dataset. We analyzed the COVID-19 data and performed data visualization, which gave a complete idea of the brief summarization of our dataset. For visualization, we used python modules like pandas, matplotlib, and seaborn. The study provided us with the summarized data using the described methods. This function prints the total distribution of the dataset, i.e., 50% of dataset, 75% of dataset, etc. We used further visualization techniques to get a better insight into our data. Using various parameters, we have analyzed our data and described the total confirmed case of COVID-19 starting from January 1 2020 to April 1 2020. We observed that as the time period increased the number of confirmed cases also increased. In Figure 1, the x-axis indicates the period as months, and the y-axis indicates the number of fatalities.

**Figure 2:**
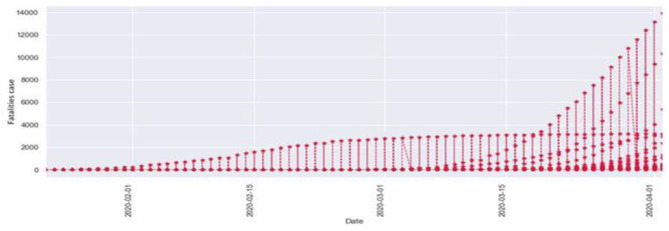
Total fatalities of COVID-19.

Figure 2 describes the total fatalities of COVID-19 starting from January. As the time period increased, the number of confirmed cases also increased. We can observe that as the time period increased at the same time the number of fatalities also increased. Here the x-axis indicates the time period in months, and the y-axis indicates the numbers of fatalities.

**Figure 3:**
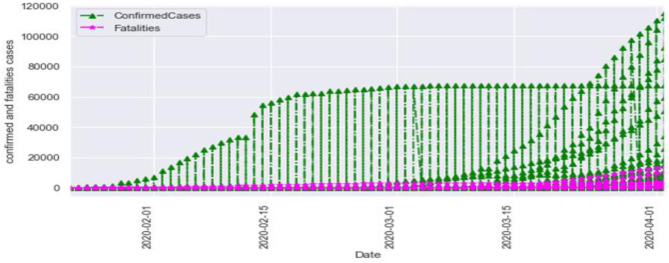
The confirmed cases vs. fatalities.

**Figure 4:**
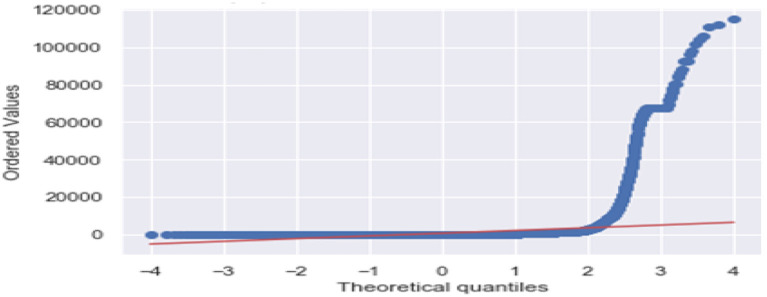
The Q-Q plot of confirmed cases.

**Figure 5:**
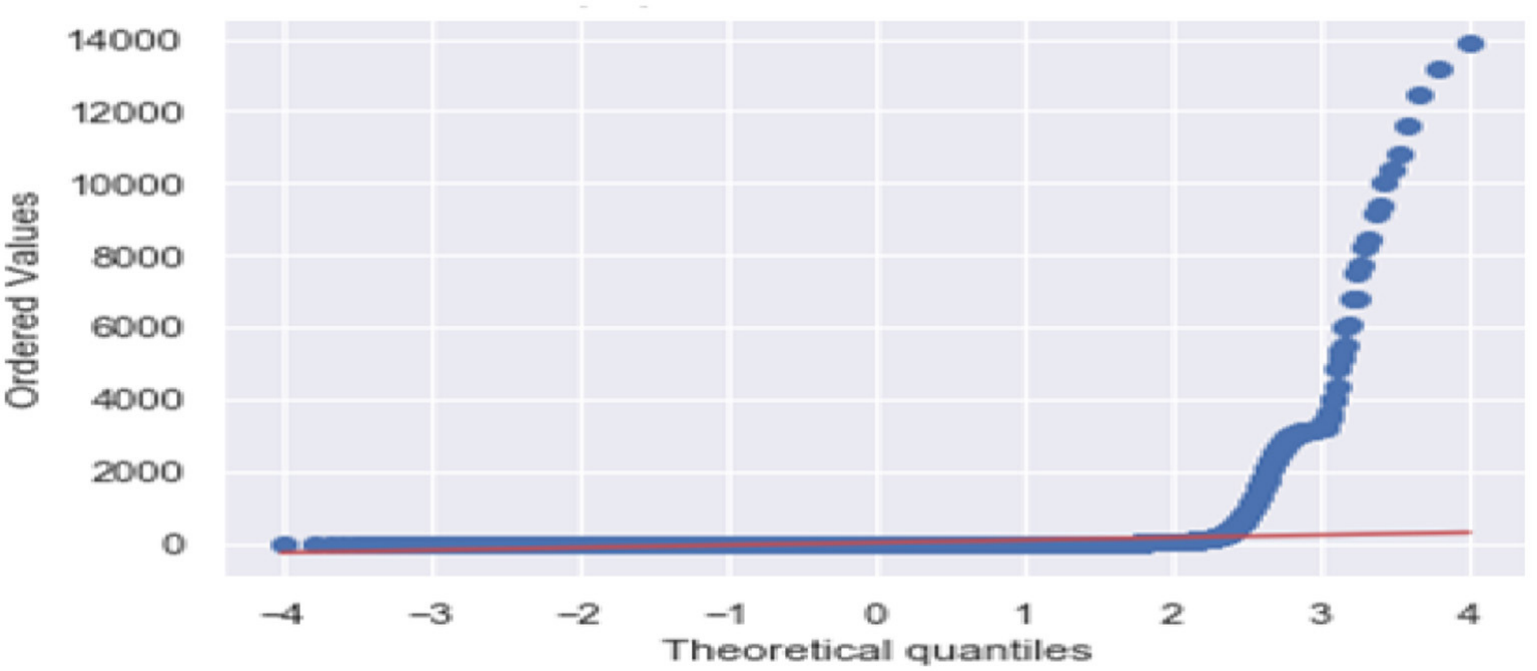
The Q-Q plot of fatalities.

Figure 3 is the comparison of increasing trends of confirmed and fatal cases over the same period of time. As we can see the legends are mentioned above in the diagram. From this graph, we observed that the number of confirmed cases were more than the number of fatalities. While the number of confirmed cases increased gradually, it was not the same for fatalities. Figures 4, 5 are referred to as Q-Q plots in statistics. These plots are a graphical technique for determining if two data sets come from populations with a standard distribution. For such modules in python, we used scipy.stats and pylab. The above Q-Q plots of confirmed cases and fatalities describe the theoretical quintiles of both cases. It means that based on the numbers and statistics, the theoretical increase in the graph should follow the red line. The quantiles-quantiles (Q-Q) plots are only used to draw the theoretical quintiles.

**Figure 6:**
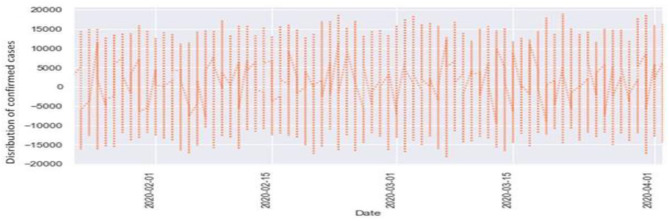
White noise time-series of confirmed cases.

**Figure 7:**
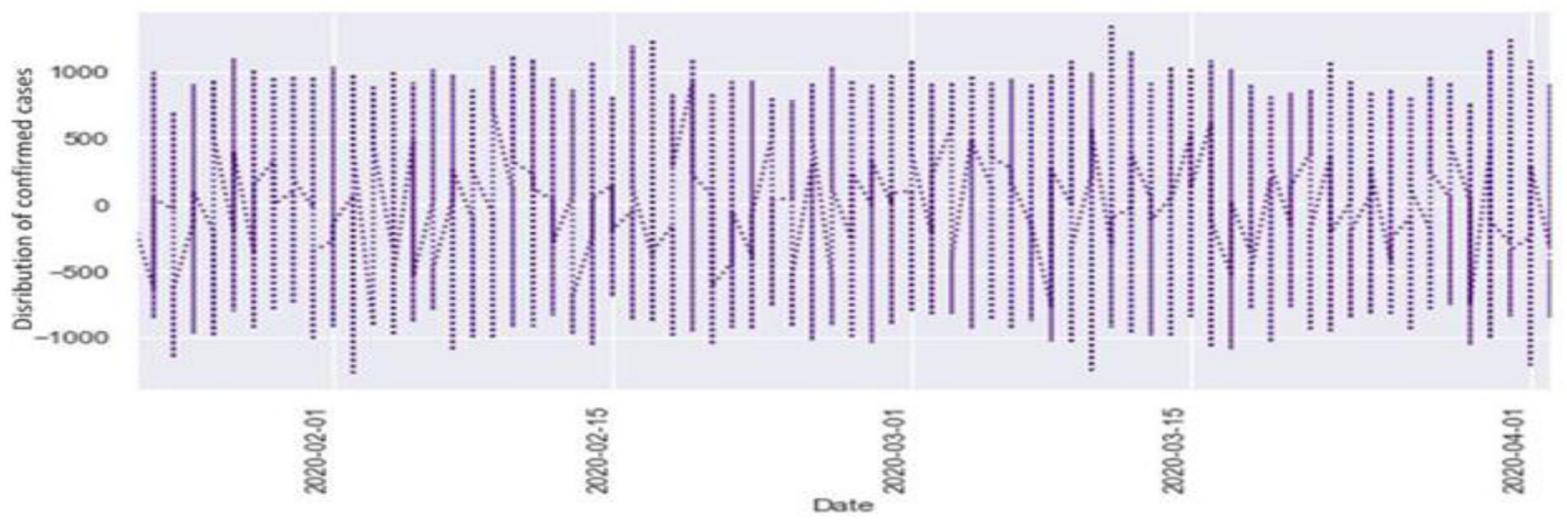
White noise time-series of fatal case.

**Figure 8:**
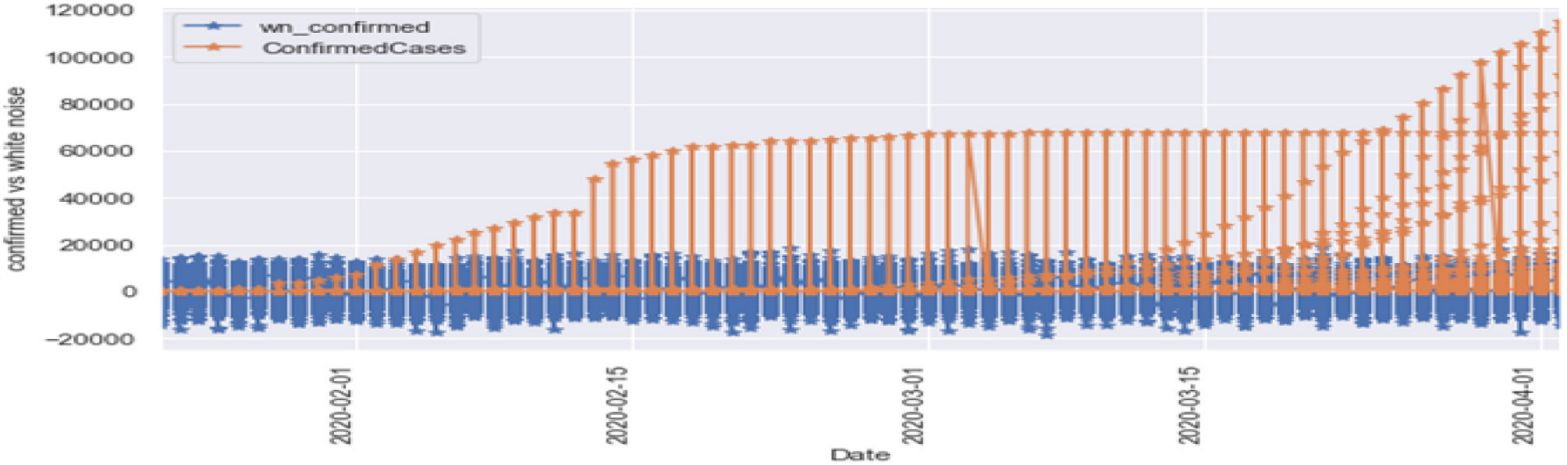
White noise confirmed cases vs. confirmed cases.

Figures 6, 7 represent the white noise time-series data of the above COVID-19 data. White noise is a sequence of independent and identically distributed random variables with finite mean and variance. Figure 6 is the white noise figure of the confirmed case and Figure 8 is the white noise figure of the fatal case. It is worth mentioning that our dataset is stationary in nature because most values are around the mean figure in the white noise data. White noise describes the particular behavior of the time-series data.

Figure 8 is the comparison of the confirmed cases and white noise data. From the graph, we can see that the initial values are mostly around white noise, which means our dataset is distributed well.

**Figure 9:**
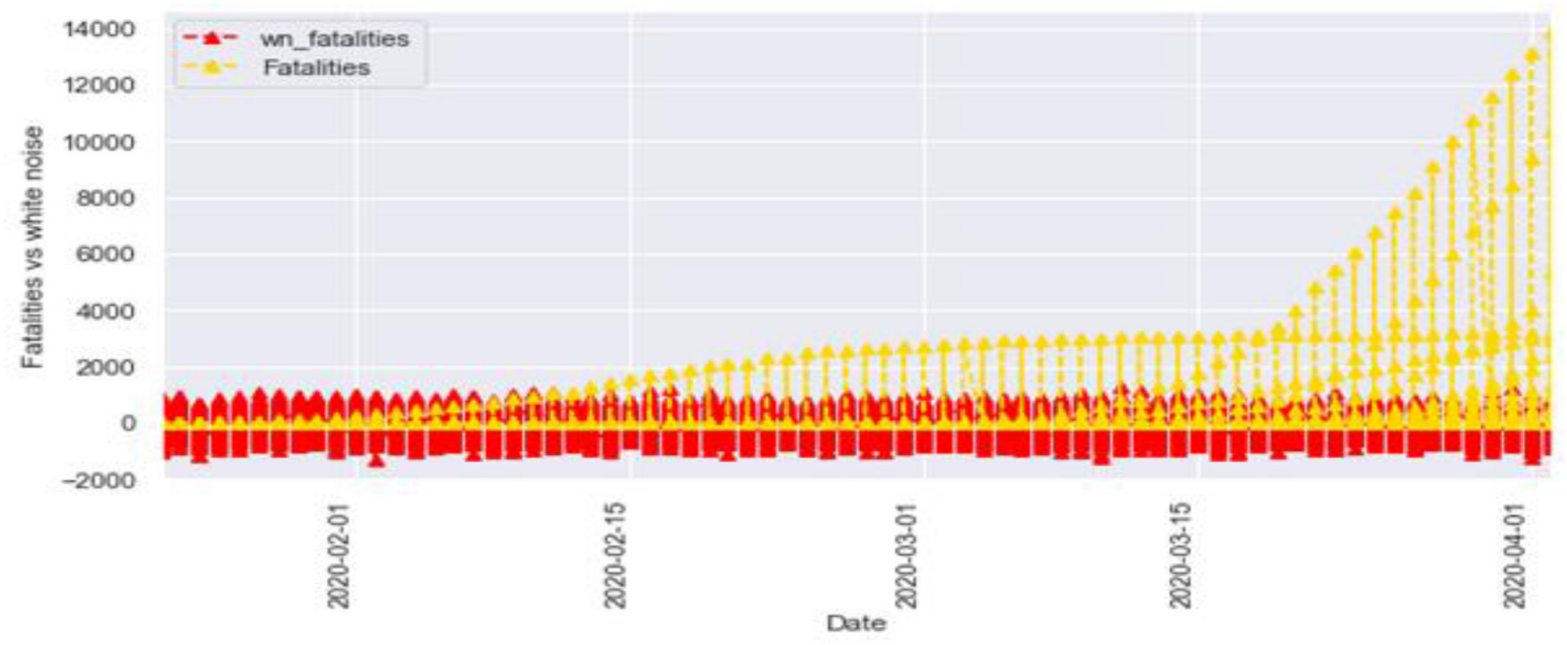
White noise fatal case vs. fatal cases.

Figure 9 represents the comparison of the fatalities and white noise data. From the graph, we can observe that most of the initial values are around the white noise, which means our dataset is distributed well.

**Figure 10:**
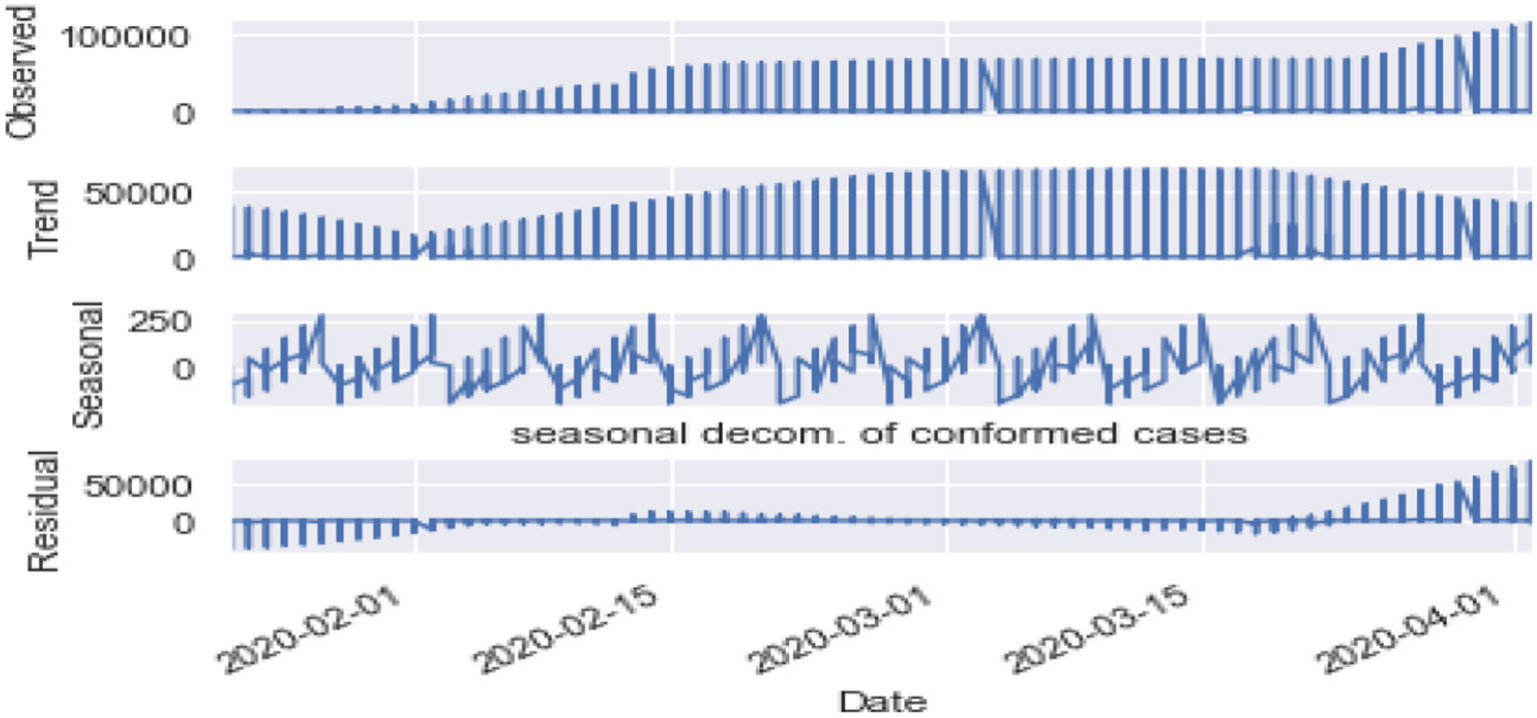
Seasonal decomposition of confirmed cases.

**Figure 11:**
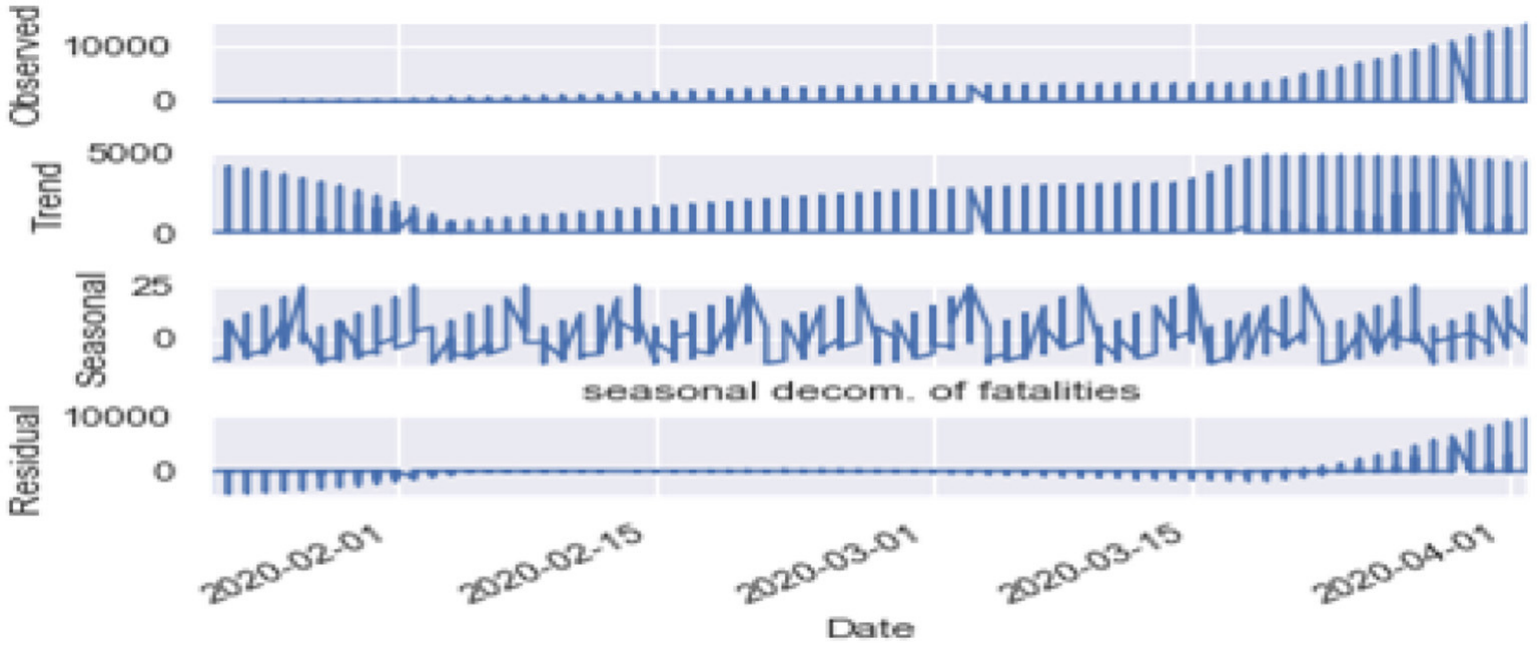
Seasonal decomposition of fatal cases.

Seasonality: Figures 10, 11 are the seasonality analysis of our data. A repeating pattern within a given time period is known as seasonality, although the term is applied more generally to repeating patterns within any fixed period. It means that we decomposed the time-series data and split them into trend, seasonal, residual, and observation. Seasonal decomposition can be performed in two ways, i.e., multiplicative. Here the term trend refers to a general systematic linear or (most often) non-linear component that changes over time and does not repeat, i.e., distribution throughout data. Seasonal refers to the cyclical effects of the dataset. Residual means the error of prediction. In a time series, it depicts what is left over after fitting a model.

**Figure 12:**
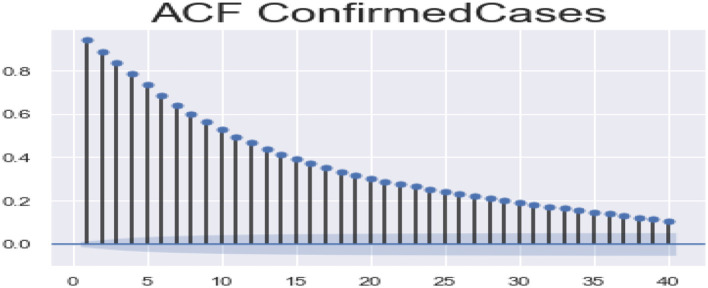
Autocorrelation function confirmed cases.

**Figure 13:**
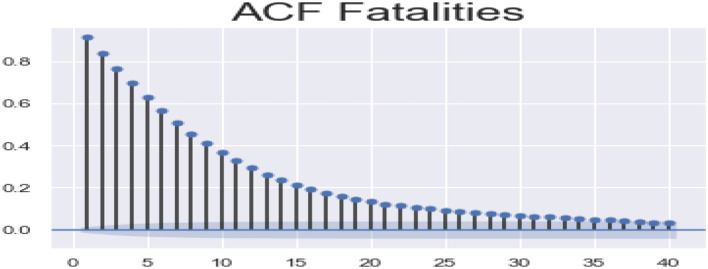
Autocorrelation function fatalities.

ACF (autocorrelation function): Figures 12, 13 depict the ACF of both confirmed cases and fatalities over time. Autocorrelation is the correlation between a sequence and itself. Statistically, it can be referred to as the correlation among the members of a variable. But in general, when the values of the observation are somehow related to each other, the corresponding stage is referred to as autocorrelation.

**Figure 14:**
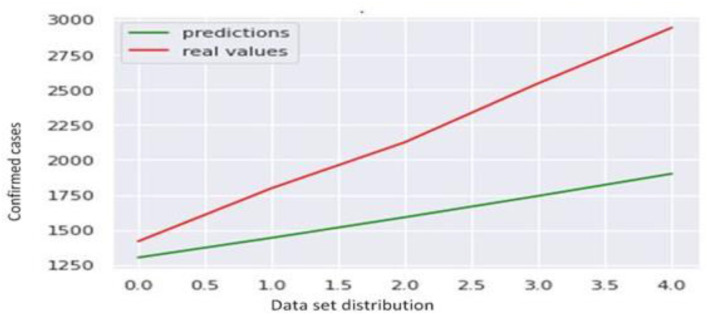
True vs. predicted values.

For model building, we have used the ARIMA model. By applying the ARIMA model, we forecast the future trend of confirmed cases and fatalities which is shown in Figure 14.

A comparative analysis of COVID-19 has been discussed in Table 2. In this study the ARIMA model focused on different global forecasting of COVID-19 total confirmed cases and total fatal cases in the earlier stage. Here in the current research work, population has been taken as a parameter.

**Table 2:** Comparative analysis.

**S/No**.	**References**	**Studied countries**	**Sources of data or repository**	**Parameters**	**Methods/model used**	**Discussion**
1	(1)	China	https://www.who.int/emergencies/diseases/novel-coronavirus-2019/situation-reports/	Fitness value	FPASSA-ANFIS	Predict the number of confirmed cases within 10 days based on previously confirmed cases
2	(25)	Italy	Italy government data	Transmission rate, recovery rate, and morality rate	ESIR	Forecasting total number COVID-19 cases
3	(26)	India (Maharashtra, Gujarat, Delhi)	https://www.mohfw.gov.in/.	Statistical parameter and metrics ETs and GEP	Genetic programming (GP)	Predict confirmed and death case
4	(27)	Brazil	World health organization	Total population of Brazil	Number of susceptible, exposed, infectious, recovered (SEIR)	Policy-making for avoiding outbreak in metropolitan cities
5	Our work	Global forecasting	https://www.kaggle.com/c/covid19-global-forecasting-week-2/data	Fraction of population	Autoregressive integrated moving average (ARIMA)	Forecast confirmed and fatalities case of COVID-19 across the globe

## Conclusion

In response to the COVID-19 pandemic, we applied time series analysis to find different measurements like the stationary, trend, and the pattern of the dataset. Various visualization techniques have been applied to the dataset for studying the outbreak related to COVID-19. We have relied on seaborn and matplotlib modules for the same. The graphs appropriately describe the trend and pattern of the COVID-19 pandemic outbreak. The time-series model ARIMA has been used to forecast the cases of COVID-19 in the future and has successfully calculated the total confirmed cases and fatalities over the studied dates and Q-Q plots of confirmed cases and fatalities. We have also estimated the total confirmed cases and fatalities over the date-Q plots. The dataset is stationary in nature; we have presented the ACF of both confirmed cases and fatalities over time and forecasted the future trend for the same. This study provides an advanced level of work, which may be useful in analyzing as well as fighting the pandemic. In future work, we can apply advanced algorithms and techniques for preparing the model which will improve and forecast more precisely.

## Data Availability Statement

The datasets presented in this study can be found in online repositories. The names of the repository/repositories and accession number(s) can be found in the article/supplementary material.

## Author Contributions

DD and RK: conceptualization and writing—original draft preparation. DD, JD, and MM: methodology. RS and RK: software. D-NL: validation and visualization. RK and RS: writing—review and editing, formal analysis, and supervision. RK: investigation. D-NL and JD: data curation. All authors: contributed to the article and approved the submitted version.

## Conflict of Interest

The authors declare that the research was conducted in the absence of any commercial or financial relationships that could be construed as a potential conflict of interest.

## References

[1] Priyadarshini I Mohanty P Kumar R Son LH Chau HTM Nhu VH. Analysis of outbreak and global impacts of the COVID-19. Healthcare. (2020) 8:148. 10.3390/healthcare8020148 32485875 PMC7349011

[2] Chen Y Liu Q Guo D. Emerging coronaviruses: genome structure, replication, and pathogenesis. J Med Virol. (2020) 92:418–23. 10.1002/jmv.25681 31967327 PMC7167049

[3] Ge XY Li JL Yang XL Chmura AA Zhu G Epstein JH. Isolation and characterization of a bat SARS-like coronavirus that uses the ACE2 receptor. Nature. (2013) 503:535–8. 10.1038/nature12711 24172901 PMC5389864

[4] Wang LF Shi Z Zhang S Field H Daszak P Eaton BT. Review of bats and SARS. Emerg. Infect Dis. (2006) 12:1834. 10.3201/eid1212.060401 17326933 PMC3291347

[5] Organization, W. H. Novel Coronavirus (2019-nCoV) (2020). Available online at: https://www.who.int/ (accessed January 27, 2020).

[6] Lu R Zhao X Li J Niu P Yang B Wu H. Genomic characterization and epidemiology of 2019 novel coronavirus: implications for virus origins and receptor binding. Lancet. (2020) 395:565–74. 10.1016/S0140-6736(20)30251-8 32007145 PMC7159086

[7] Cauchemez S Van Kerkhove M Riley S Donnelly C Fraser C Ferguson N. Transmission scenarios for Middle East Respiratory Syndrome Coronavirus (MERS-CoV) and how to tell them apart. Euro Surveill. (2013) 18:20503. 23787162 PMC4088931

[8] Chan JF Yuan S Kok KH To KK Chu H Yang J. A familial cluster of pneumonia associated with the 2019 novel coronavirus indicating person-to-person transmission: a study of a family cluster. Lancet. (2020) 395:30154–9. 10.1016/S0140-6736(20)30154-9 31986261 PMC7159286

[9] Otter JA Donskey C Yezli S Douthwaite S Goldenberg SD Weber DJ. Transmission of SARS and MERS coronaviruses and influenza virus in healthcare settings: the possible role of dry surface contamination. J Hosp Infect. (2016) 92:235e50. 10.1016/j.jhin.2015.08.027 26597631 PMC7114921

[10] Dowell SF Simmerman JM Erdman DD Wu JS Chaovavanich A Javadi M. Severe acute respiratory syndrome coronavirus on hospital surfaces. Clin Infect Dis. (2004) 39:652e7. 10.1086/422652 15356778 PMC7107915

[11] Geller C Varbanov M Duval RE. Human coronaviruses: insights into environmental resistance and its influence on the development of new antiseptic strategies. Viruses. (2012) 4:3044e68. 10.3390/v4113044 23202515 PMC3509683

[12] Kampf G Antiseptic Stewardship: Biocide Resistance and Clinical Implications. Cham: Springer International Publishing (2018). p. 78–84. 10.1007/978-3-319-98785-9

[13] Cheng ZJ Shan J. Novel Coronavirus: Where we are and What We Know. Infection. (2019) 48:155–63. 10.1007/s15010-020-01401-y 32072569 PMC7095345

[14] Guan WJ Ni ZY Hu Y Liang WH Ou CQ He JX Clinical characteristics of 2019 novel coronavirus infection in China. medRxiv. (2020) 382:1708–20. 10.1101/2020.02.06.20020974 PMC7092819 32109013

[15] Shaman J Karspeck A. Forecasting seasonal outbreaks of influenza. Proc Natl Acad Sci USA. (2012) 109:20425–30. 10.1073/pnas.1208772109 23184969 PMC3528592

[16] Jang JS. ANFIS: adaptive-network-based fuzzy inference system. IEEE Trans. Syst. Man Cybern. (1993) 23:665–85. 10.1109/21.25654118249858

[17] Liu Q Liu X Jiang B Yang W. Forecasting incidence of hemorrhagic fever with renal syndrome in China using ARIMA model. BMC Infect Dis. (2011) 11:218. 10.1186/1471-2334-11-218 21838933 PMC3169483

[18] Scarpino SV Petri G. On the predictability of infectious disease outbreaks. Nat Commun. (2019) 10:47–54. 10.1038/s41467-019-08616-0 30796206 PMC6385200

[19] Chowell G Luo R Sun K Roosa K Tariq A Viboud C. Real-time forecasting of epidemic trajectories using computational dynamic ensembles. Epidemics. (2020) 30:100379. 10.1016/j.epidem.2019.100379 31887571

[20] Tian CW Wang H Luo XM. Time-series modelling and forecasting of hand, foot and mouth disease cases in China from 2008 to 2018. Epidemiol Infect. (2019) 147:1–3. 10.1017/S095026881800362X 30868999 PMC6518604

[21] Zeng Q Li D Huang G Xia J Wang X Zhang Y. Time series analysis of temporal trends in the pertussis incidence in Mainland China from 2005 to 2016. Sci Reports. (2016) 6:32367. 10.1038/srep32367 27577101 PMC5006025

[22] Ture M Kurt I Comparison of four different time series methods to forecast hepatitis A virus infection. Expert Systems Applications. (2006) 31:41–6. 10.1016/j.eswa.2005.09.002

[23] Massad E Burattini MN Lopez LF Coutinho FA. Forecasting versus projection models in epidemiology: the case of the SARS epidemics. Med. Hypotheses. (2005) 65:17–22. 10.1016/j.mehy.2004.09.029 15893110 PMC7116954

[24] Shaman J Yang W Kandula S. Inference and forecast of the current West African Ebola outbreak in Guinea, Sierra Leone and Liberia. PLoS Curr. (2014) 6:1–17 10.1371/currents.outbreaks.3408774290b1a0f2dd7cae877c8b8ff6 25642378 PMC4234409

[25] Jia W Han K Song Y Cao W Wang S Yang S. Extended SIR prediction of the epidemics trend of COVID-19 in Italy and compared with Hunan, China. medRxiv. (2020) 7:1–7. 10.1101/2020.03.18.20038570 32435645 PMC7218168

[26] Salgotra R Gandomi M Gandomi AH. Time series analysis and forecast of the COVID-19 pandemic in India using genetic programming. Chaos Solitons Fractals. (2020) 138:109945. 10.1016/j.chaos.2020.109945 32508399 PMC7260529

[27] Rocha Filho TM dos Santos FSG Gomes VB Rocha TA Croda JH Ramalho WM Expected impact‘ of COVID-19 outbreak in a major metropolitan area in Brazil. medRxiv. (2020) 14:20035873 10.1101/2020.03.14.20035873

